# Clinicopathological relevance of tumor expression of NK group 2 member D ligands in resected non-small cell lung cancer

**DOI:** 10.18632/oncotarget.27308

**Published:** 2019-11-26

**Authors:** Riki Okita, Ai Maeda, Katsuhiko Shimizu, Yuji Nojima, Shinsuke Saisho, Masao Nakata

**Affiliations:** ^1^Department of General Thoracic Surgery, Kawasaki Medical School, Kurashiki, Japan

**Keywords:** non-small cell lung cancer (NSCLC), UL16-binding protein (ULBP), prognostic factor, MICA/B (MHC class I chain-related molecule A and B), NK cell

## Abstract

UL16-binding protein (ULBP) 1-6 and MHC class I chain-related molecule A and B (MICA/B) are NK group 2, member D (NKG2D) ligands, which are specifically expressed in infected or transformed cells and are recognized by NK cells via NKG2D-NKG2D ligand interactions. We previously reported that MICA/B overexpression predicted improved clinical outcomes in patients with resected non-small cell lung cancer (NSCLC). However, the clinicopathological features and prognostic significance of ULBPs in NSCLC remain unclear. Here,ULBP1-6 expression was evaluated based on immunohistochemistry of 91 NSCLC samples from patients following radical surgery. ULBPs were expressed by the majority of NSCLC. Either ULBP1 or ULBP2/5/6 overexpression was associated with squamous-cell carcinoma histology, whereas ULBP4 overexpression was associated with younger age and adenocarcinoma histology. Although overexpression of ULBP1-6 did not impact clinical outcomes in NSCLC patients, integrative profiling with cluster analysis classified patients into 3 subgroups based on the expression pattern of NKG2D ligands. The subgroup characterized by ULBP1 or ULBP2/5/6 high expressing but ULBP4 low expressing tumors showed poor overall survival. Taken together with previous results, NSCLC histological subtype strongly correlates with NKG2D ligands expression pattern. NKG2D ligands expression levels assessed by multiple immune parameters could predict clinical outcomes of patients with NSCLC.

## INTRODUCTION

Lung cancer is the leading cause of cancer-related deaths worldwide [1]. Although TNM classification is the principal guide used for the prognostic evaluation of non-small cell lung cancer (NSCLC) [2], several immunological factors such as tumor infiltrating lymphocytes [3] or molecules related to immune-recognition such as human leukocyte antigen-A2 [4] are also suitable in order to predict the clinical outcome of patients with NSCLC. The use of immune checkpoint inhibitors is considered to be a highly effective therapeutic strategy for patients with NSCLC [5, 6], which prompted interest in evaluating the expression of T cell-related immunological factors such as Programmed cell death-1 (PD-1) ligand 1 (PD-L1) both in tumor cells and immune cells [7]. On the other hand, the role of NK cell-related immunological factors in NSCLC remains unclear, although NK cell count is a predictive factor for clinical benefit of PD-1 targeted therapy in melanoma [8].

The main role of NK cells is considered to be immunosurveillance [9]. NK group 2, member D (NKG2D) ligands consist of MHC class I chain-related molecule A and B (MICA/B) and UL16-binding protein (ULBP) 1-6, which promote NK cell-mediated cytotoxicity via the NK cell activating receptor NKG2D [10] and are expressed in transformed or infected cells [11]. Hypothetically, NK cell-mediated cytotoxicity against tumor cells should be enhanced if the NKG2D ligand is overexpressed in tumor cells. Indeed, NKG2D ligand overexpression was reported to be correlated with a better prognosis in several types of cancer [12–15]. In NSCLC, we previously reported that overexpression of MICA/B predicted improved clinical outcomes for resected NSCLC patients [16]. However, there is no report evaluating the correlation between ULBP1-6 expression and clinical outcome in patients with NSCLC, although high concentrations of serum-soluble ULBP2 in NSCLC patients were reported to be correlated with poor prognosis [17].

In this study, we evaluated the expression of ULBP1-6 using immunohistochemistry for samples from patients with resected, pathological stage (pStage) IA-IIIA NSCLC, using the same dataset as for our previous “MICA/B” study [16], and assessed the relationship between the expression status of each ULBP and patient characteristics or clinical outcomes. Additionally, previously described data from the “MICA/B” study [16] was updated in order to compare the clinical impact of MICA/B expression status with that of ULBPs. Our results showed that ULBP1 and ULBP2/5/6 are predominantly expressed in lung squamous cell carcinoma, while ULBP4 is expressed in lung adenocarcinoma. These findings suggest that ULBP1 and ULBP2/5/6 are promising targets for the treatment of lung squamous cell carcinoma while ULBP4 is one for the treatment of lung adenocarcinoma. Although overexpression of ULBPs has less impact on the survival of patients with resected NSCLC than overexpression of MICA/B, cluster analysis showed that the subgroup which was characterized by ULBP1 or ULBP2/5/6 high expressing but ULBP4 low expressing tumors showed poor overall survival.

## RESULTS

### Relationship between ULBP expression and clinical characteristics of NSCLC

**Figure 1:**
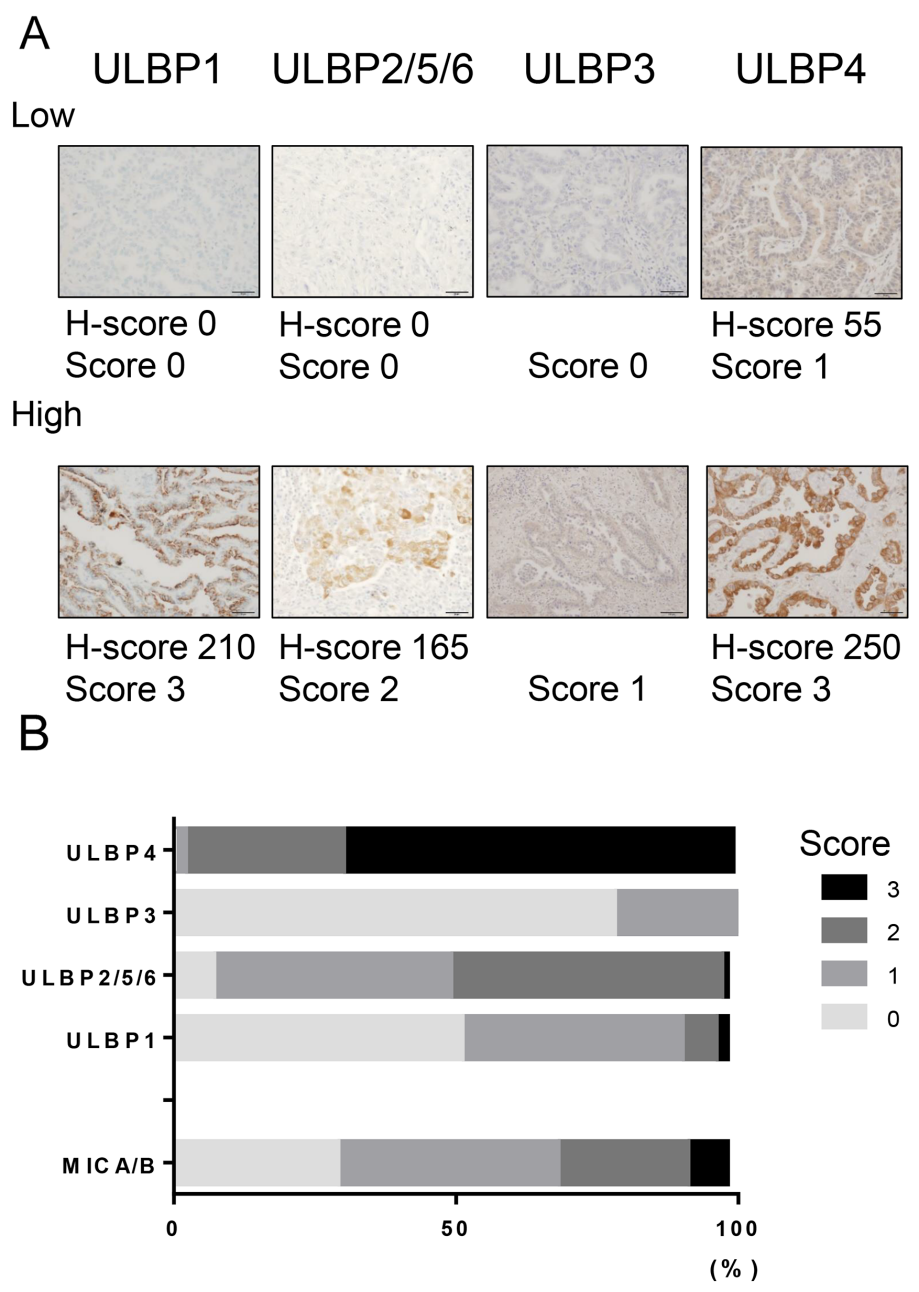
NKG2D ligand expression in NSCLC tissues **(A)** Immunohistochemical staining of ULBPs in NSCLC tissues. Representative staining for ULBP1, ULBP2/5/6, ULBP3, and ULBP4 in tumor tissues (200× magnification). The panels show images corresponding to different intensity scores of NKG2D ligand expression. Low: score 0-1 for ULBP1, -2/5/6, -4 or score 0 for ULBP3. High; score 2-3 for ULBP1, -2/5/6, -4 or score 1-3 for ULBP3. **(B)** Frequency of NKG2D ligand expression in non-small cell lung cancer. The graph shows the expression levels of each NKG2D ligand.

Representative immunohistochemical stains for ULBPs are shown in Figure 1A. The expected cut-off values for the score of each molecule according to Receiver operating characteristic (ROC) curves (Supplementary Figure 1) are as follows: ULBP1: Score 1, ULBP2/5/6 Score 0: ULBP3: score 0, and ULBP4: score 2. From a total of 91 tumors, overexpression of ULBP1, ULBP2/5/6, ULBP3, and ULBP4 was found in 44 (48.4%), 45 (49.5%), 20 (22.0%), and 63 (69.2%) cases, respectively (Figure 1B), while that of MICA/B was found in 28 (30.8%) cases, as previously described [16]. The clinicopathological characteristics of NSCLC samples are summarized in Supplementary Table 1. Interestingly, either ULBP1 or ULBP2/5/6 overexpression was correlated with a squamous cell carcinoma histology, while ULBP4 overexpression was correlated with younger age and adenocarcinoma histology (Table 1).

**Table 1:** Clinicopathological characteristics by NKG2D ligand

Characteristic	Number (n=91)		ULBP1			ULBP2/5/6			ULBP3			ULBP4		
			low	high	*p*	low	high	*p*	low	high	*p*	low	high	*p*
Age	67.8 (37-83)		66.6±8.6	69.1±8.1	0.165	68.8±7.2	66.7±9.4	0.241	67.7±8.2	68.1±9.4	0.872	**71.3±7.1**	**66.2±8.5**	**0.007** ^*^
Gender	Male	59 (65%)	27	32	0.127	26	33	0.093	48	11	0.297	20	39	0.380
	Female	32 (35%)	20	12		20	12		23	9		8	24	
Smoking^a^	Never	33 (37%)	20	13	0.170	20	13	0.126	25	8	0.726	8	25	0.364
	Current or former	57 (63%)	26	31		25	32		45	12		19	38	
CEA (ng/ml)			11.6±35.4	6.4±11.3	0.355	5.6±10.4	12.6±36.3	0.208	9.9±30.1	4.2±4.0	0.404	5.0±3.3	10.8±31.9	0.337
SUVmax			8.2±6.1	6.5±4.3	0.126	7.7±5.7	7.1±4.9	0.604	7.1±5.5	6.6±4.8	0.708	6.8±5.8	7.6±5.1	0.469
Tumor size (mm)			30.1±13.0	30.7±13.5	0.818	28.8±11.1	32.0±15.0	0.240	31.2±13.8	27.5±10.7	0.261	31.6±13.9	29.9±12.9	0.562
Histology	Adenocarcinoma	71 (78%)	**41**	**30**	**0.028** ^*^	**40**	**31**	**0.037** ^*^	54	17	0.394	**18**	**53**	**0.035** ^*^
	Squamous cell carcinoma	20 (22%)	**6**	**14**		**6**	**14**		17	3		**10**	**10**	
Histlogic grade	G1	38 (42%)	20	18	0.611	24	14	0.126	30	8	0.264	9	29	0.327
	G2	29 (32%)	13	16		12	17		20	9		9	20	
	G3	24 (26%)	14	10		10	14		21	3		10	14	
Pleural invasion	Negative	59 (65%)	30	29	0.836	33	26	0.163	46	13	0.986	19	40	0.687
	Positive	32 (35%)	17	15		13	19		25	7		9	23	
Lymphatic invasion	Negative	66 (73%)	36	30	0.369	34	32	0.765	51	15	0.779	20	46	0.876
	Positive	25 (27%)	11	14		12	13		20	5		8	17	
Vascular invasion	Negative	54 (59%)	27	27	0.704	30	24	0.249	43	11	0.655	13	41	0.095
	Positive	37 (41%)	20	17		16	21		28	9		15	22	
Lymphnode metastasis	N0	72 (79%)	38	34	0.675	38	34	0.408	58	14	0.256	23	49	0.636
	N1-2	19 (21%)	9	10		8	11		13	6		5	14	
Pathological stage	IA	35 (38%)	20	15	0.407	21	14	0.154	26	9	0.496	11	24	0.914
	IB-IIIA	56 (62%)	27	29		25	31		45	11		17	39	

a: Data not available for one patient.

CEA: carcinoembryonic antigen, SUVmax: maximum standardized uptake value.

* *p*<0.05

### Recurrence free survival (RFS) and overall survival (OS) stratified by ULBP expression status in resected NSCLC

**Figure 2:**
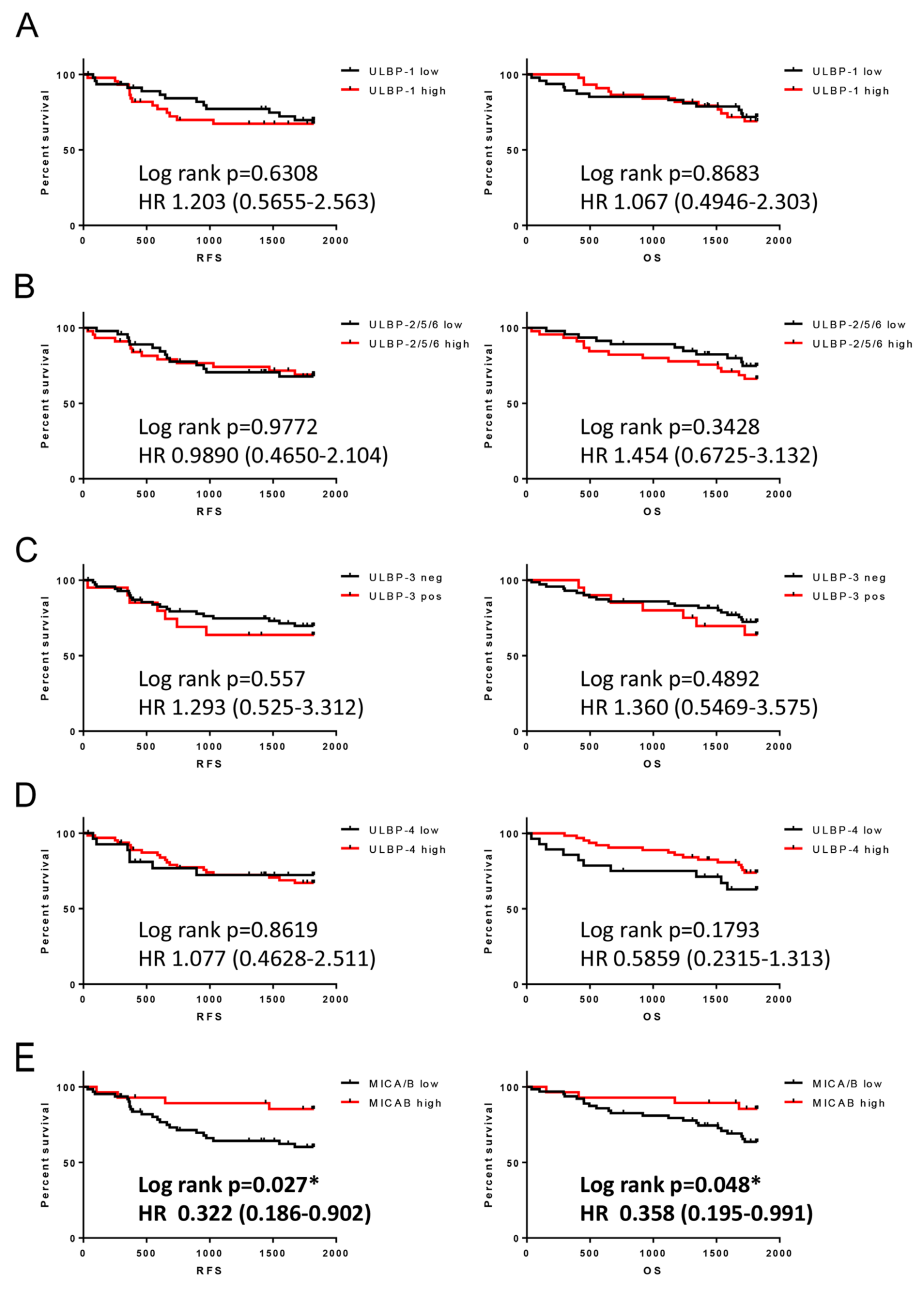
Association of NKG2D ligand expression in NSCLC with RFS and OS Kaplan-Meier plots showing RFS or OS in patients with lower or higher expression of **(A)** ULBP1, **(B)** ULBP2/5/6, **(C)** ULBP3, and **(D)** ULBP4, **(E)** updated data for MICA/B. HR: Hazard ratio. ^*^ *p* < 0.05

In order to investigate the correlation between ULBP expression and clinical outcomes, both RFS and OS were stratified using ULBP expression and evaluated. The results indicate that ULBP overexpression does not have an effect on RFS or OS (Figure 2A–D), while MICA/B overexpression was correlated with improved outcomes in both RFS and OS (Figure 2E). Although the original report showed that MICA/B overexpression could be used to predict improved outcomes in only RFS [16], if the follow-up period was updated to be the same length as that for ULBP, the effect on OS was found.

### RFS and OS stratified by multiple immune parameters in resected NSCLC

**Figure 3:**
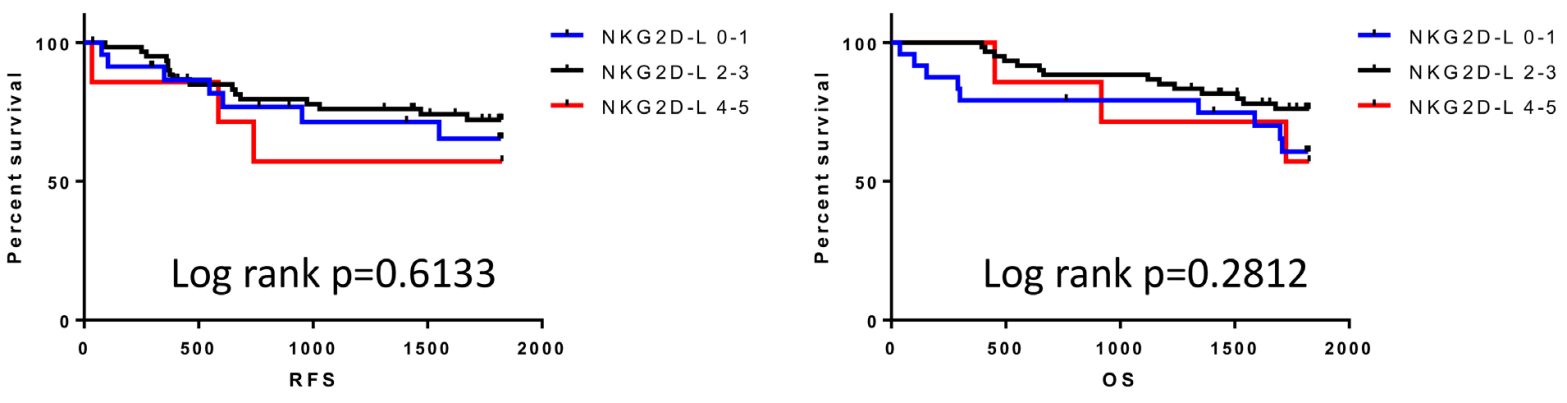
Survival outcomes for patients classified by the number of overexpressing NKG2D ligands NKG2D-L 0-1: none or one NKG2D ligand expressing tumor, NKG2D-L 2-3: 2 or 3 NKG2D ligand expressing tumor, NKG2D-L 4-5: 4 or 5 NKG2D ligand expressing tumor.

**Figure 4:**
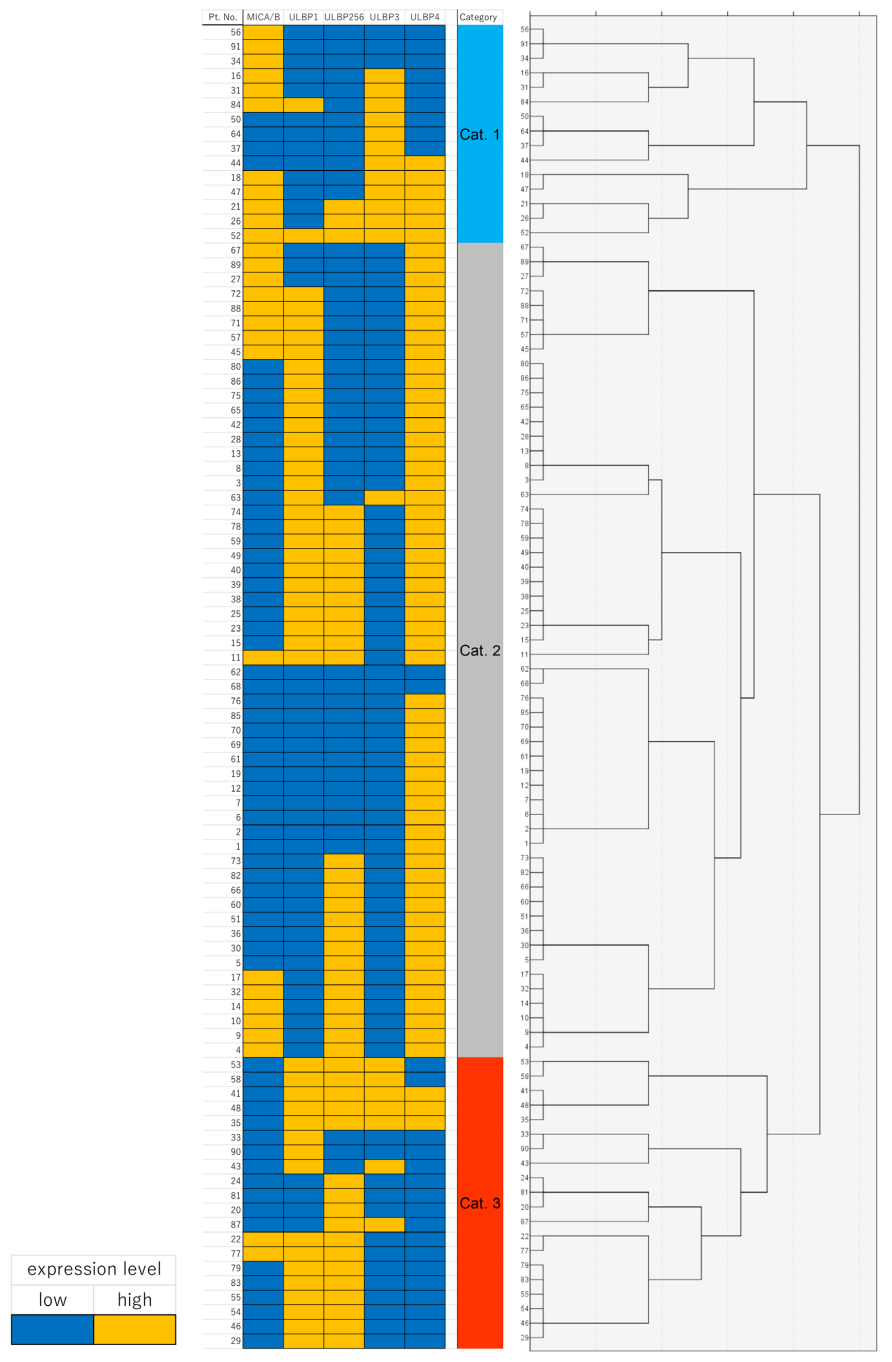
Heat map of immunohistochemical protein expression of MICA/B, ULBP1, ULBP2/5/6, ULBP3, and ULBP4 in the cluster map The consensus matrix is used as the similarity matrix to define the final clusters.

**Figure 5:**
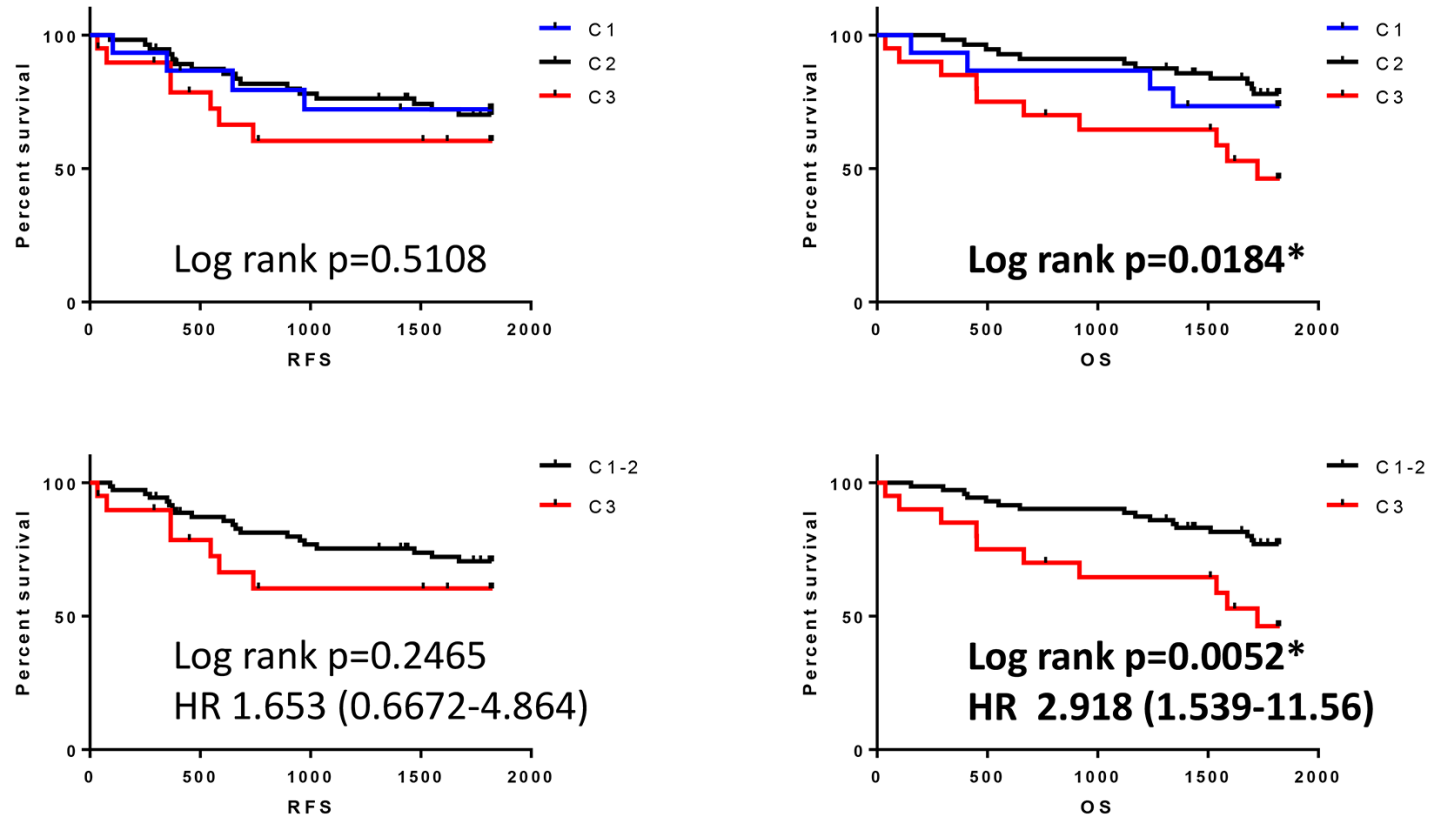
Survival outcomes in patients classified by cluster analysis C1: category 1, C2: category 2, C3: category 3, C1-2: Category 1 or 2. HR: Hazard ratio. ^*^ *p* < 0.05

In order to assess the impact of the overexpression of multiple NKG2D ligands on clinical outcome, RFS and OS were stratified by determining the number of overexpressed NKG2D ligands. Unexpectedly, the number of overexpressed NKG2D ligands had no impact on either RFS or OS (Figure 3). To assess the multiple immune parameters further, integrative profiling with cluster analysis classified our patients into 3 subgroups (category 1, 2, and 3) based on the expression pattern of NKG2D ligands, including MICA/B (Figure 4). Interestingly, category 3, the subgroup which was mainly characterized by ULBP1 or ULBP2/5/6 high expressing but ULBP4 low expressing tumors, showed poor OS compared with category 1 or 2, although there was no impact on RFS (Figure 5).

### Category 3 immune parameters were independent prognostic factors for poor outcome in resected NSCLC

Cox regression analysis was performed to determine the predictive value of clinical variables for RFS and OS. Univariate analysis showed that lymphatic invasion, vascular invasion, and lymph node metastasis were potential predictors of RFS. Multivariate analysis showed only lymph node metastasis to be a prognostic factor for poor outcome (hazard ratio (HR) 4.779, *p*=0.009) for RFS (Table 2). On the other hand, univariate analysis showed that pleural invasion, lymphatic invasion, vascular invasion, lymph node metastasis, and the category were potential predictors of OS. Surprisingly, multivariate analysis showed the category to be an independent prognostic factor for poor clinical outcome (HR 0.329, *p*=0.009), while lymph node metastasis was not a significant prognostic factor (Table 3).

**Table 2:** Cox proportional hazard model for RFS (n=91)

	Univariate		Multivariate	
	Hazard ratio (95% CI)	*p* value	Hazard ratio (95% CI)	*p* value
Gender (male vs female)	0.787 (0.353-1.753)	0.558	0.833 (0.195-3.566)	0.806
Smoking^a^ (non- vs smoker)	1.107 (0.507-2.418)	0.799	1.266 (0.265-6.044)	0.767
Histology (Ad vs Sq)	1.153 (0.436-3.045)	0.775	2.083 (0.714-6.077)	0.179
Pleural invasion (neg vs pos)	1.723 (0.806-3.682)	0.160	1.305 (0.458-3.718)	0.618
Lymphatic invasion (neg vs pos)	4.940 (2.295-10.631)	**<0.001** ^*^	1.787 (0.533-6.000)	0.347
Vascular invasion (neg vs pos)	2.721 (1.643-4.506)	**<0.001** ^*^	1.178 (0517-2.685)	0.697
Lymphnode metastasis (neg vs pos)	7.852 (3.606-17.100)	**<0.001** ^*^	4.779 (1.487-15.361)	**0.009** ^*^
Category (1,2 vs 3)	0.604 (0.255-1.4312)	0.252	0.580 (0.236-1.425)	0.235

a: Data not available for one patient.

RFS: recurrence free survival, CI: confidence interval, Ad: adenocarcinoma, Sq: squamous cell carcinoma.

*: *p*>0.05

**Table 3:** Cox proportional hazard model for OS (n=91)

	Univariate		Multivariate	
	Hazard ratio (95% CI)	*p* value	Hazard ratio (95% CI)	*p* value
Gender (male vs female)	0.583 (0.245-1.387)	0.223	0.778 (0.162-3.734)	0.753
Smoking^a^ (non- vs smoker)	1.812 (0.762-4.313)	0.179	1.516 (0.300-7.661)	0.615
Histology (Ad vs Sq)	0.847 (0.340-2.110)	0.722	1.451 (0.522-4.029)	0.475
Pleural invasion (neg vs pos)	2.476 (1.145-5.356)	**0.021** ^*^	2.034 (0.776-5.334)	0.149
Lymphatic invasion (neg vs pos)	3.011 (1.392-6.512)	**0.005** ^*^	1.503 (0.490-4.612)	0.476
Vascular invasion (neg vs pos)	2.180 (1.305-3.644)	**0.003** ^*^	1.088 (0.519-2.279)	0.824
Lymphnode metastasis (neg vs pos)	3.737 (1.710-8.166)	**0.001** ^*^	2.643 (0.919-7.603)	0.071
Category (1,2 vs 3)	0.341 (0.154-0.753)	**0.008** ^*^	0.329 (0.144-0.753)	**0.009** ^*^

a : Data not available for one patient.

OS: Overall survival, CI: confidence interval, Ad: adenocarcinoma, Sq: squamous cell carcinoma.

* : *p*>0.05

## DISCUSSION

Recent developments in the use of immune checkpoint inhibitors as treatments have improved prognosis of advanced NSCLC [5, 6]. Nevertheless, 60-70% of patients receiving immune checkpoint therapy tend to develop tumor progression, which requires additional strategies for NSCLC treatment. NK cells play an important role in host immunity against the tumor, participating mainly in immune surveillance [9]. Recently, it was reported that NK cell count before PD-1/PD-L1 targeted therapy predicted treatment response in patients with melanoma [8]. Moreover, NK cell count decreased after regulatory T cell-depletion therapy using the anti-CCR4 monoclonal antibody Mogamulizumab [20], suggesting that NK cells also affect immune checkpoint therapy. However, the clinical significance of NK cells in immune checkpoint targeted therapy remains unclear. In this study, we have shown that approximately half or more of NSCLC cases express ULBP1, ULBP-2/5/6 or ULBP4, while less than a quarter of cases express ULBP3. Interestingly, ULBP1 or ULBP2/5/6 tend to be overexpressed in lung squamous cell carcinoma, while ULBP4 is largely overexpressed in lung adenocarcinoma. We previously showed that MICA/B overexpression in NSCLC cells was independently associated with a good prognosis in terms of RFS, and the updated data showed that MICA/B overexpression is useful for the prediction of improved clinical outcomes in terms of both RFS and OS. On the other hand, overexpression of ULBP1-6 did not show any impact on either RFS or OS. Moreover, our result showed that the number of overexpressed NKG2D ligands had no impact on clinical outcomes, although several studies showed that ULBP overexpression could predict better prognosis in patients with breast cancer [12], cervical cancer [13], and hepatocellular carcinoma [14, 15]. Following the assessment of multiple immune parameters among NKG2D ligands, cluster analysis showed that a subgroup, which was mainly characterized by either ULBP1 or ULBP2/5/6 high expressing but ULBP4 low expressing tumors, showed poor overall survival. Surprisingly, category 3 characteristics are a stronger prognostic factor than lymph node metastasis in terms of OS, suggesting that further study of multiple immune parameters could be a more useful classification than the TNM staging system.

The present study could also provide some ideas for the development of ULBP targeted therapies. Our data suggested that an antibody type drug targeting ULBP1 or ULBP2/5/6 might be appropriate for the treatment of squamous cell carcinoma whereas one targeting ULBP4 might be useful for treating adenocarcinoma, as more than a half of studied tumors expressed each of these molecules, respectively. Another promising strategy is NKG2D expressing Chimeric Antigen Receptor engineered (CAR) cell therapy [21, 22]. NKG2D expressing CAR cells could recognize the tumor cells which express at least one NKG2D ligand on their surface, independent of the type of NKG2D ligand. The present study found only 2 cases (2.2%) with an all negative phenotype of NKG2D ligand expression, suggesting that over 90% of tumors might be good targets for NKG2D-CAR cell therapy.

In conclusion, we have investigated the expression pattern of ULBP1-6 in resected NSCLC. Although the ULBP expression pattern has no impact on the prognosis of NSCLC, histological subtypes are strongly correlated with ULBP expression pattern. To develop an NKG2D ligand targeted therapy using monoclonal antibodies, patients should be selected by considering the tumor histological subtype or the expression status of the NKG2D ligand. Moreover, NKG2D expressing CAR-T or CAR-NK cells have good potential for the treatment of both lung adenocarcinoma and lung squamous cell carcinoma. The TNM system is well established for predicting clinical outcomes. However, cluster analysis for classification via multiple immune parameters might be a superior predictive system if the number of immune parameters is increased.

## MATERIALS AND METHODS

### Patients and specimens

The present study was approved by the Kawasaki Medical School ethics committee (No. 1227-4) and written informed consent was obtained from all patients before surgery for the use of resected specimens. Enrollment criteria of the patients and routine post-operative check-up details were previously described [16]. To compare the impact of MICA/B and ULBPs on the clinical outcome of patients with NSCLC, the follow-up period for MICA/B was extended from our previous study [16] to match the length of follow-up for ULBPs. Patients characteristics are shown in Supplementary Table 1.

### Immunohistochemical staining

Our study included formaldehyde-fixed, paraffin-embedded NSCLC specimens which were collected from pStage IA-IIIA patients. ULBP1, ULBP3, and ULBP4 expression was determined by performing immunohistochemical staining for tissue samples from NSCLC patients using a mouse monoclonal anti-ULBP1 antibody (clone 3F1, Santa Cruz), anti-ULBP-3 antibody (clone D-1, Santa Cruz), and an anti-ULBP-4 antibody (clone #709116, R&D systems), respectively, according to a previously described protocol [16, 18]. ULBP2/5/6 expression was evaluated by performing immunohistochemical staining using a goat anti-ULBP2/5/6 polyclonal antibody (R&D systems) and anti-Goat HRP-DAB Cell & Tissue Staining Kit (R&D systems). Mesothelioma or lung cancer tissue was used as a positive control for MICA/B, ULBP1, ULBP2/5/6, ULBP4, while bronchus was used as an internal control for ULBP3. The primary antibody was omitted from the negative control (Supplementary Table 2). Tissue slides were counterstained with hematoxylin, following which they were examined by two investigators who had no prior knowledge of the corresponding clinicopathological data. Cytosolic or membrane intensity of immunoreactivity was scored by the investigators. The intensity scoring for staining was defined as follows: “0”: no staining, “1+”: weak staining that was visible only with high magnification, “2+”: moderate staining (between 1+ and 3+), and “3+”: strong staining that was visible with low magnification. The histoscore (H-score) was calculated according to the following formula: 1 × (%cells 1+) + 2 × (%cells 2+) + 3 × (%cells 3+) [19]. The expression levels of ULBPs were defined as follows: “Score 0”: H-score 0, “Score 1”: H-score 1-99, “Score 2”: H-score 100-199, “Score 3”: H-score 200-300.

### Statistical analysis

ROC curves for ULBPs in order to predict NSCLC recurrence were generated to determine the expected cut-off value that yielded optimal sensitivity and specificity. Chi-square tests or Fischer exact tests were performed to evaluate the relationship between ULBP expression levels and patient characteristics. Kaplan-Meier survival analysis was used to determine the association between ULBP expression and RFS or OS until death or last follow-up; the significance of the differences in RFS or OS between groups was assessed by log-rank test using GraphPad Prism 6.01 (GraphPad Software, La Jolla, CA). Cluster analysis used for the classification of our patients into subgroups based on the expression pattern of NKG2D ligands including MICA/B was performed using SPSS statistical package 17.0 (SPSS, Chicago, IL). Univariate and multivariate analyses were performed using the Cox proportional hazards model in order to identify independent prognostic factors. Statistical analyses were also performed using the SPSS statistical package 17.0. In all cases, *p* < 0.05 was considered significant. The follow up period was set to a maximum of 5 years (1825 days). The median length of follow up was 1522 days (range, 37 to 1825 days) for all patients and the last follow-up date was October 6, 2017.

## SUPPLEMENTARY MATERIALS FIGURES AND TABLES



## Abbreviations

Ad: Adenocarcinoma
CAR-T: Chimeric Antigen Receptor engineered (CAR) T cell
CAR-NK: Chimeric Antigen Receptor engineered (CAR) NK cell
CEA: Carcinoembryonic antigen
CI: Confidence interval
FDG-PET/CT: ^18^F-fluorodeoxyglucose positron emission tomography/computed tomography
HR: Hazard ratio
MICA/B: MHC class I chain-related molecule A and B
NKG2D: NK group 2, member D
NSCLC: Non-small-cell lung cancer
OS: Overall survival
PD-1: Programmed cell death-1
PD-L1: Programmed cell death-1 ligand 1
ROC: Receiver operating characteristic
RFS: Recurrence free survival
Sq: Squamous cell carcinoma
SUVmax: Maximum standard uptake value
ULBP: UL16-binding protein
