# Supplementary material for: Clinicopathological relevance of tumor expression of NK group 2 member D ligands in resected non-small cell lung cancer
Source: Oncotarget. 2019 Nov 26;10(63):6805–15. doi: 10.18632/oncotarget.27308 (PMC6887580; doi:10.18632/oncotarget.27308)
Supplement: Supplementary file 1 [file oncotarget-10-6805-s001.pdf]

# Clinicopathological relevance of tumor expression of NK group 2 member D ligands in resected non-small cell lung cancer

## SUPPLEMENTARY MATERIALS

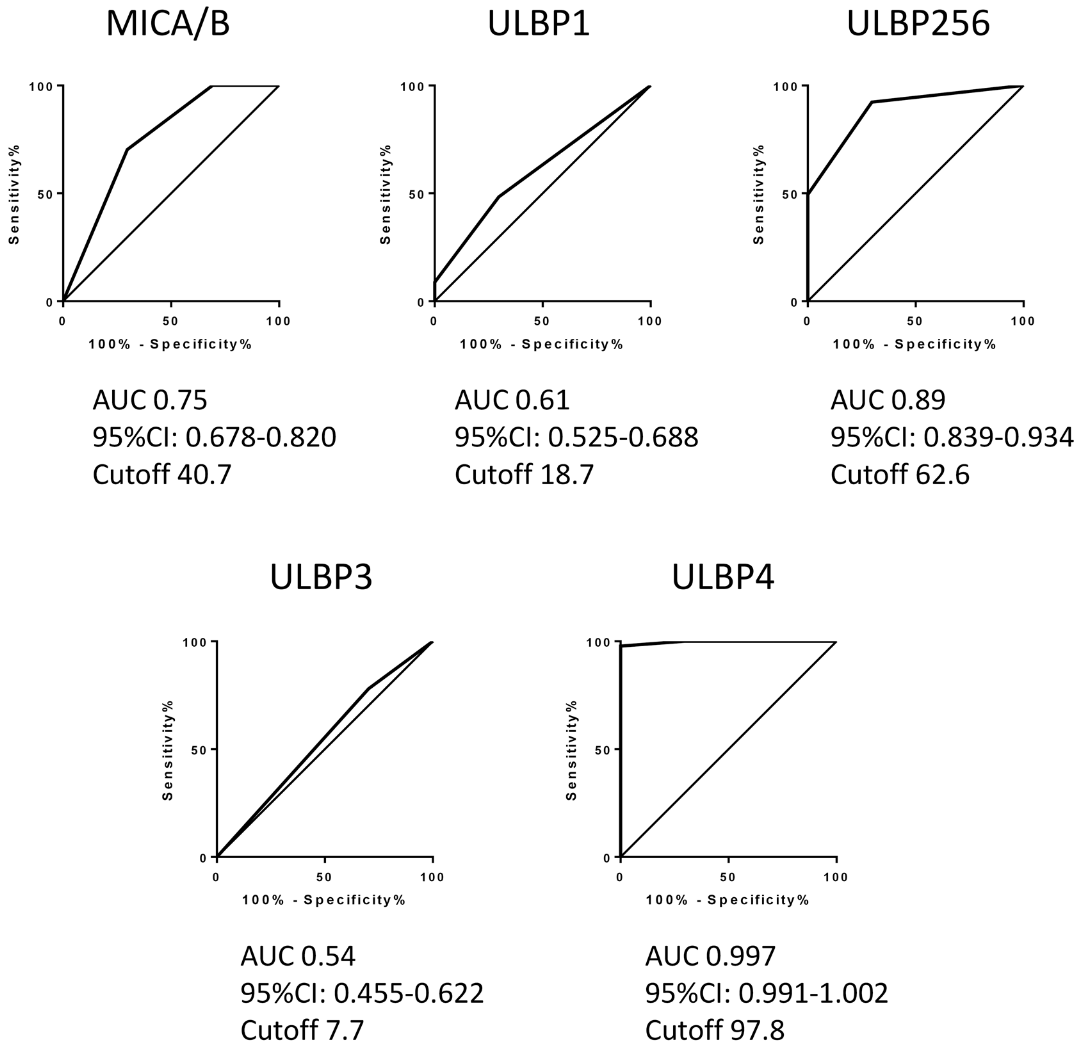


**Supplementary Figure 1: Receiver operating characteristics (ROC) curve for predicting recurrence free survival.** AUC: Area under the curve, CI: Confidence interval.

**Supplementary Table 1: Patient and tumor characteristics (n=91)**

| | |
|---|---|
| Age, years (range) | 67.8 (37-83) |
| Gender (male/female) | 59/32 |
| Smorking (never/current or former)[a] | 33/57 |
| Histology (Ad/Sq) | 71/20 |
| Pleural invasion (0/1-3) | 59/32 |
| Lymphatic invasion (0/1-3) | 66/25 |
| Vascular invasion (0/1-3) | 54/37 |
| Lymphnode metastasis (N0/N1-3) | 72/19 |
| Pathological Stage (IA/IB-IIIA) | 35/56 |
| Postoperative Recurrence (−/+) | 64/27 |

[a]: Data not available for one patient.

**Supplementary Table 2: Antibodies used in immunohistochemical reaction**

<table>
<tr><th rowspan="2">Antigen</th><th rowspan="2">Species</th><th rowspan="2">Clonality</th><th rowspan="2">Clone</th><th rowspan="2">Catalog number</th><th rowspan="2">Vendor</th><th rowspan="2">Dilution</th><th colspan="2">Antigen retrival condition</th><th>negative</th><th>Positive</th></tr>
<tr><th>method</th><th>Retrival solution</th><th>control</th><th>control</th></tr>
<tr><td>MICA/B</td><td>mouse</td><td>mono</td><td>D-8</td><td>sc-271535</td><td>Santa Cruz</td><td>1:50</td><td rowspan="5">heating the slides 3 times at 100°C</td><td rowspan="5">citrate buffer (pH6)</td><td rowspan="5">omit primary Ab</td><td>MPM tissue</td></tr>
<tr><td>ULBP1</td><td>mouse</td><td>mono</td><td>3F1</td><td>sc-53131</td><td>Santa Cruz</td><td>1:100</td><td>LC tissue</td></tr>
<tr><td>ULBP2/5/6</td><td>goat</td><td>poly</td><td>-</td><td>AF1298</td><td>R&D systems</td><td>1:20</td><td>MPM tissue</td></tr>
<tr><td>ULBP3</td><td>mouse</td><td>mono</td><td>D-1</td><td>sc-390844</td><td>Santa Cruz</td><td>1:100</td><td>bronchus (internal control)</td></tr>
<tr><td>ULBP4</td><td>mouse</td><td>mono</td><td>#709116</td><td>MAB6285</td><td>R&D systems</td><td>1:200</td><td>LC tissue</td></tr>
</table>

Ab: antibody, MPM: malignant pleural mesothelioma, LC: lung cancer
